# Patient-Reported Outcomes and Preferences for Colon Capsule Endoscopy and Colonoscopy: A Systematic Review with Meta-Analysis

**DOI:** 10.3390/diagnostics11091730

**Published:** 2021-09-20

**Authors:** Ulrik Deding, Pablo Cortegoso Valdivia, Anastasios Koulaouzidis, Gunnar Baatrup, Ervin Toth, Cristiano Spada, Ignacio Fernández-Urién, Marco Pennazio, Thomas Bjørsum-Meyer

**Affiliations:** 1 Department of Clinical Research, University of Southern Denmark, 5230 Odense, Denmark; gunnar.baatrup@rsyd.dk (G.B.); thomas.bjoersum-meyer@rsyd.dk (T.B.-M.); 2 Department of Surgery, Odense University Hospital, 5000 Odense, Denmark; 3 Gastroenterology and Endoscopy Unit, University Hospital of Parma, University of Parma, 43121 Parma, Italy; cortegosopablo@yahoo.it; 4 Department of Social Medicine & Public Health, Faculty of Health Sciences, Pomeranian Medical University, 70-204 Szczecin, Poland; akoulaouzidis@hotmail.com; 5 Department of Gastroenterology, Skåne University Hospital, Lund University, 221 00 Malmö, Sweden; ervin.toth@med.lu.se; 6 Digestive Endoscopy Unit, Fondazione Poliambulanza Istituto Ospedaliero, 25133 Brescia, Italy; cristianospada@gmail.com; 7 Department of Gastroenterology, Complejo Hospitalario de Navarra, 31008 Pamplona, Spain; ifurien@yahoo.se; 8 University Division of Gastroenterology, AOU Città Della Salute e Della Scienza, University of Turin, 10124 Turin, Italy; pennazio.marco@gmail.com

**Keywords:** colonoscopy, colon capsule endoscopy, patient-reported outcomes, endoscopy

## Abstract

Colon capsule endoscopy as an alternative to colonoscopy for the diagnosis of colonic disease may serve as a less invasive and more tolerable investigation for patients. Our aim was to examine patient-reported outcomes for colon capsule endoscopy compared to conventional optical colonoscopy including preference of investigation modality, tolerability and adverse events. A systematic literature search was conducted in Web of Science, PubMed and Embase. Search results were thoroughly screened for in- and exclusion criteria. Included studies underwent assessment of transparency and completeness, after which, data for meta-analysis were extracted. Pooled estimates of patient preference were calculated and heterogeneity was examined including univariate meta-regressions. Patient-reported tolerability and adverse events were reviewed. Out of fourteen included studies, twelve had investigated patient-reported outcomes in patients who had undergone both investigations, whereas in two the patients were randomized between investigations. Pooled patient preferences were estimated to be 52% (CI 95%: 41–63%) for colon capsule endoscopy and 45% (CI 95%: 33–57%) for conventional colonoscopy: not indicating a significant difference. Procedural adverse events were rarely reported by patients for either investigation. The tolerability was high for both colon capsule endoscopy and conventional colonoscopy. Patient preferences for conventional colonoscopy and colon capsule endoscopy were not significantly different. Procedural adverse events were rare and the tolerability for colon capsule endoscopy was consistently reported higher or equal to that of conventional colonoscopy.

## 1. Introduction

Early-stage detection and removal of colorectal pre-cancerous polyps is an effective measure for the prevention of colorectal cancer (CRC) [1]. To date, conventional optical colonoscopy (COC) is the current reference standard for examining the mucosal lining. However, in 5–20% of patients referred for COC, the procedure is incomplete due to insufficient bowel cleansing or patient intolerance [2,3,4]. Capsule endoscopy (CE) technology was initially developed to study the small bowel (SB) mucosa [5]. The SB capsule proved to be exceptionally tolerated by patients, which boosted further research in developing capsules for other parts of the gastrointestinal (GI) tract. In 2006, the first generation of the colon capsule endoscope (CCE1), equipped with a twin set of cameras, was introduced as a promising diagnostic alternative to COC [6]. Due to mediocre results in large trials, the CCE1 was replaced by the improved second-generation CCE (CCE2), currently in use, with reported adenoma detection rates equal to COC [7,8]. However, the lack of steerability and camera focus control does no extra favors to CCE. In the wake of the COVID-19 pandemic, the technology is being increasingly adopted in clinical practice in England and Scotland to ease overburdened healthcare systems [9,10]. Conversely, CCE may be performed as an out-patient procedure with minimal involvement of healthcare professionals; moreover, the incidence of severe adverse events (AE) is very low. Patients undergoing CCE have been reported with less discomfort and embarrassment in many studies, juxtaposed against COC [11].

The existing knowledge on patient discomfort and minor AE with CCE is scarce as patients only have renewed contact with the hospital if a subsequent COC is mandatory and, even so, patient-reported outcomes (PRO) are rarely addressed. Patient acceptance and preference towards CCE are of outermost importance for a broader clinical implementation. Hence, we find it relevant to conduct a systematic review and meta-analysis to collect and present existing data on PRO and the preference for CCE compared to COC.

## 2. Materials and Methods

### 2.1. Data Sources and Search Strategy

We conducted a systematic literature search in PubMed, Embase and Web of Science to identify all relevant citations for studies in which acceptability, tolerance or preference was expressed by the patients (i.e., PRO), after COC and CCE were performed. The primary outcome of our analyses were the preference proportions, whereas acceptability and tolerance were secondary outcomes. According to the PICO search strategy, search terms were included in three areas: investigation, comparator and outcome. The terms within each area were combined using the Boolean “OR”; the three areas were combined in strings using the Boolean “AND”. Free text search terms with truncation were included. The literature search was concluded on 12 January 2021. The complete search strings are available in Appendix A. The study was registered at the PROSPERO international register of systematic reviews (ID: CRD42021231718).

### 2.2. Inclusion and Exclusion Criteria

Inclusion criteria were:

- full text articles;
- articles reporting PRO after undergoing both COC and CCE or randomized controlled trials (RCT);
- articles in English/Danish/Italian/French/Spanish language.

Exclusion criteria were:

- non-RCT articles in which patients underwent either COC or CCE alone;
- the following article types: reviews, conference papers, case reports.

### 2.3. Screening of References

After identification and exclusion of duplicates, references were independently screened by three authors (U.D., P.C.V., T.B.-M.). Each author screened two thirds of the references in title and abstract, excluding those not meeting the inclusion criteria. In case of discrepancy, the reference was included for full text evaluation. After this first step, the process was repeated on included references by evaluating full texts with the same modality.

### 2.4. Data Extraction

All data were extracted in accordance with the Preferred Reporting Items for Systematic Reviews and Meta-Analyses (PRISMA) [12]. We collected demographic data of the patients, the setting for intervention, the type of CE (CCE1, CCE2 or PillCam^®^ Crohn), cleanliness and completion rates for both COC and CCE. COC procedures were considered complete when the caecum was reached; CE procedures were considered complete when the CE was excreted during the recording time or when the anal verge or the hemorrhoidal plexus were visualized. PRO and AE were also collected. Regarding PRO, the preference for COC and CCE was expressed as proportions (%). AE were limited to events related to the procedure itself, excluding those related to bowel preparation (BP).

### 2.5. Study Assessment and Risk of Bias

A study assessment of the included studies was performed by three independent reviewers (UD, PCV, TBM) through the STROBE assessment tool and the Cochrane Collaboration’s tool (according to the study design) [13,14]. STROBE items 6b, 12d, 14c and 16c were omitted as they were not applicable to the included studies.

For STROBE, we arbitrarily designated studies as of low-, medium- and high transparency and completeness according to the tools’ outcome scores: for STROBE, the cut-offs were respectively 60% and 80% of the total maximum points (*n* = 30). For Cochrane, within each bias category (selection, performance, detection, attrition, reporting), the bias level was rated as low, high or unclear.

Although aware that the primary aim of STROBE and Cochrane tools is to improve the overall quality of reporting, we empirically assumed that the higher number of points in STROBE and higher number of low risk of bias assessments in Cochrane, the higher the transparency and completeness of the included study.

### 2.6. Statistical Analysis

To evaluate the proportion of patients preferring CCE or COC, respectively, we calculated the preference proportions of CCE and COC. This was defined as the proportion of patients preferring each out of the total number of patients responding. Patients responding to data collection (interview or questionnaire) but not indicating preference were included in the denominator. Non-responders were excluded. The significance level was set at 5%, and 95% confidence intervals (CI) were calculated. All pooled estimates were calculated in random effects models using the Freeman–Tukey double arcsine transformation. In sensitivity analyses, we repeated the preference proportions calculations, first, after exclusion of studies from which only a subset of the sample could be included, and second, after exclusion of studies with samples under 50 individuals. I^2^ statistics were performed to test and evaluate the heterogeneity by applying thresholds provided by the Cochrane Handbook [15]. Potential sources of heterogeneity were tested by univariate meta-regressions. To investigate publication bias and small study effects, Egger’s tests [16] were performed and illustrated by funnel plot. Individual study data were extracted and compiled in spreadsheets for pooled analyses. Data analyses were conducted using Stata 16 (StataCorp, College Station, TX, USA, 2019. Stata Statistical Software: Release 16, StataCorp LLC) including the metaprop command [17].

## 3. Results

### 3.1. Search

**Figure 1:**
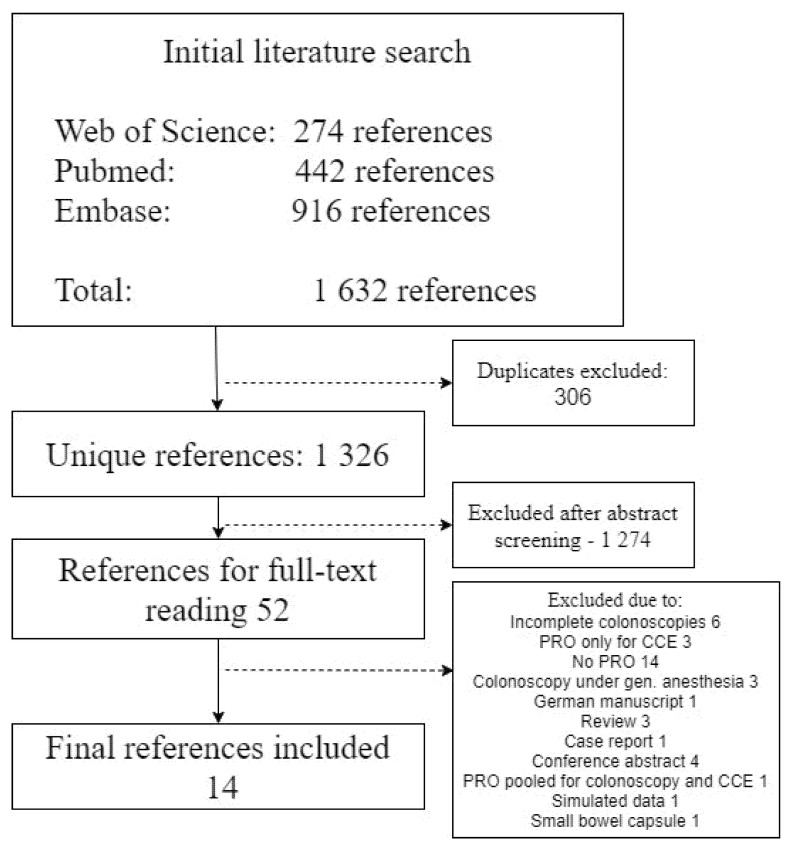
Consort diagram of the systematic review. Abbreviations: CCE, colon capsule endoscopy; PRO, patient-reported outcomes.

The initial literature search resulted in 1632 references. After the removal of duplicates this was reduced to 1326 and, additionally, 1274 were excluded after title and abstract screening. Full text screening was performed for 52 references and 14 were eventually included in the study (Figure 1) [11,18,19,20,21,22,23,24,25,26,27,28,29,30]. In 12 studies, participants had undergone both CCE and COC, and in two (*n* = 2) studies they were randomized for either. Included studies are described in Table 1. Ten (*n* = 10) studies reported patient preference for CCE and eight (*n* = 8) reported patient preference for COC. Four studies did not report patient preference but described patient-reported AE and/or tolerability.

Nine (*n* = 9) studies were evaluated as having high transparency and completeness and five (*n* = 5) studies as medium, see Appendix B.

### 3.2. Patient Preference

**Figure 2:**
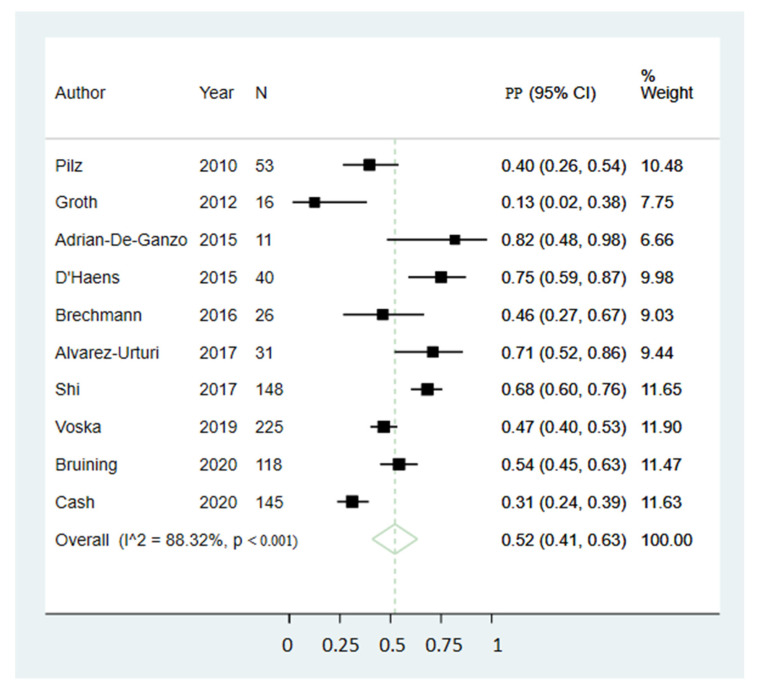
Pooled estimate of CCE preference proportion. Abbreviations: CI, confidence interval; PP, Estimated preference proportion.

**Figure 3:**
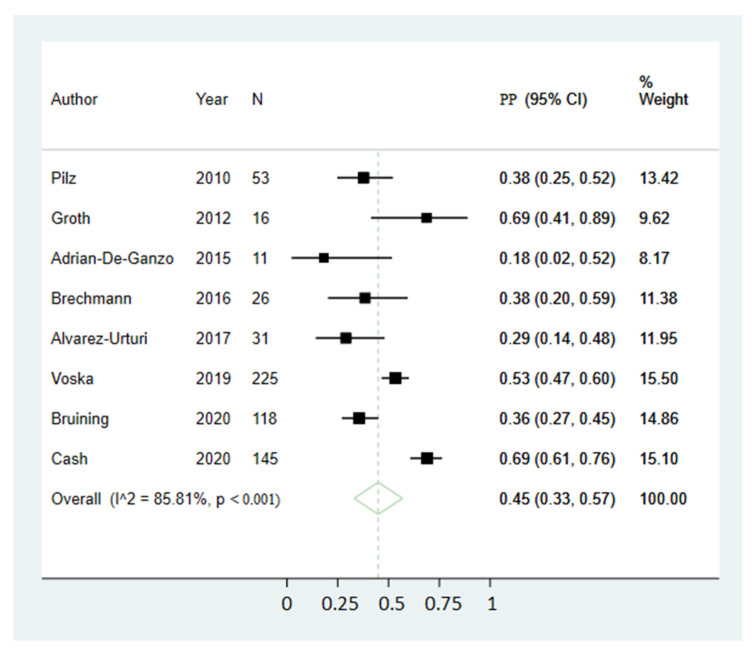
Pooled estimate of colonoscopy preference proportion. Abbreviations: CI, confidence interval; PP, Estimated preference proportion.

Pooled preference estimates were 52% (95% CI 41;63) (Figure 2) for CCE and 45% (95% CI 33;57) (Figure 3) for COC, not indicating a significant difference. Preference for CCE varied between studies from 13% to 82%, whereas the range for COC preference was 18% to 69%. Reported reasons for preferring CCE included less invasiveness, no need for sedation or driver, only one investigation for all GI segments, no need for intravenous access, less embarrassment and discomfort, the mobility and access to investigation and, finally, fear of COC-related discomfort or complications. Reported reasons for preferring COC included it being a familiar procedure, the opportunity to perform biopsies, less time-consuming, less BP, it being a standard procedure and that CCE restricts daily life activities. The sensitivity analysis excluding studies with only a subset population showed similar estimates where the pooled preference estimates were 54% (95% CI 42;65) for CCE and 45% (95% CI 32;58) for COC, not indicating a significant difference. The sensitivity analysis excluding small size studies showed similar estimates where the pooled preference estimates were 48% (95% CI 35;61) for CCE and 49% (95% CI 35;64) for COC, not indicating a significant difference.

**Figure 4:**
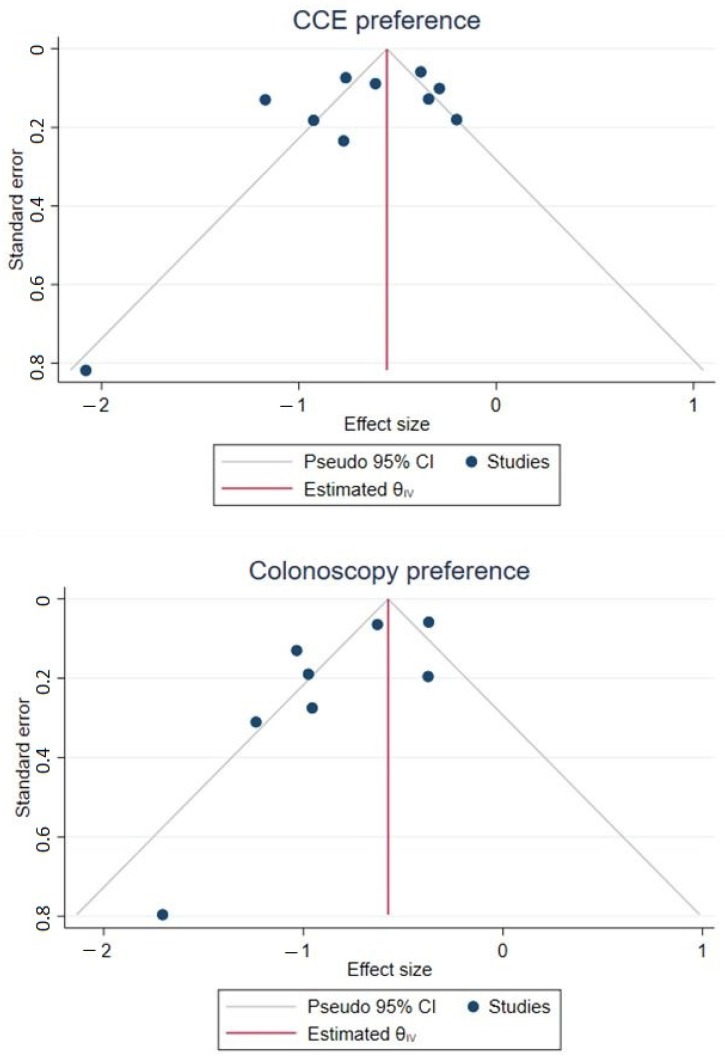
Funnel plots.

As substantial heterogeneity was present in both preference proportion estimations (I^2^ = 88.32% and 85.81), univariate meta-regressions were performed to identify possible sources. None of the tested variables resulted in statistically significant effects, although age mean yielded the lowest observed *p*-values for both CCE and COC preference analyses (Table 2). Funnel plots are included (Figure 4) illustrating possible publication bias. Egger’s tests for small study effects were performed for each pooled preference proportion estimation with *p*-values of 0.084 for CCE and 0.040 for COC, indicating the presence of small study effects, at least for the COC estimate.

### 3.3. Adverse Events

Very low proportions of AE (often none) were reported in the included studies. Only one (*n* = 1) moderate/severe AE (i.e., capsule retention) was reported in CCE from patients. More retentions could be expected as several of the included studies report retrieving capsules in the following colonoscopy if not excreted. Mild patient-reported AE from CCE included difficulty with camera ingestion and abdominal discomfort. In comparison, two (*n* = 2) moderate/severe AE in COC were reported from patients (i.e., colonic perforation). Mild patient-reported AE from COC included local phlebitis due to intravenous access, abdominal pain, rectal bleeding and elevated blood pressure. In conclusion, moderate to severe procedural AE were rarely reported by patients for both CCE and COC.

### 3.4. Tolerability

Several studies reported results that were collected using visual analog scales (VAS) and/or numerical scales. Median overall rating reported from 1 (very bad) to 10 (very good) was 8 for CCE and 7 for COC in 148 individuals [29]. On a numerical scale ranging from 1 to 10, 238 patients reported their discomfort level from COC and 239 from CCE. Low level discomfort (<4) was reported by 35.2% for colonoscopy versus 88.5% for CCE. High level discomfort was reported by 27.2% for COC versus 0.4% for CCE [11]. Using a five-point scale rating from none through mild, moderate, severe and intolerable, the embarrassment was rated severe or worse in 39% for COC and 8% for CCE by 40 patients. For discomfort this was 42% for COC and 11% for CCE [23]. Thirteen (*n* = 13) ulcerative colitis patients rated overall tolerability for CCE and COC on a ten-point scale to be 7.9 average for both procedures, even though they rated abdominal pain 4.9 for COC and 1.0 for CCE on the same scale [27]. Similar results were reported from 32 patients with previous colorectal surgery who reported a mean tolerance score of 8.50 for CCE and 8.56 for COC on a ten-point VAS. The same group rated comfort of the procedure to be 8.56 for CCE and 8.63 for COC [24]. In 31 individuals with familial CRC, everyone rated CCE to be good or better, compared to 84% for colonoscopy, on a five-point scale (poor, fair, good, very good and excellent) [19].

## 4. Discussion

Our systematic review with meta-analysis shows no significant difference in patients’ preference between COC and CCE. About half of the included patients, having tried both modalities, declare an equal preference for the two colonic imaging modalities. One would have expected that patient preference would sway towards CCE as it can provide a virtually painless exploration of the colon, with similar diagnostic yield and accuracy to COC in many clinical situations. Nevertheless, all that glitters is not gold: as highlighted in the results of our study, the watershed of PRO for CCE versus COC remains blurred; the stigma surrounding colonoscopy, often raising well-known barriers in the CRC screening populations, failed to show any apparent inclination towards CCE as one may have expected, concerning PRO. On the other hand, a substantial proportion of patients still preferred CCE even though COC is considered the golden standard. The reality may be that each investigation should target the patient in question and not be considered a one-test-fits-all strategy.

In a recent interim analysis [31], we asked a screening population, before receiving their result from a submitted fecal immunochemical test, to indicate which investigation they would prefer. In 14,461 individuals, 50.0% chose CCE and 9.9% preferred COC, while those remaining did not mark a preference. The preference for CCE before the investigation compared to after seems similar (i.e., as in the present study), while the preference for COC is much higher after the procedure, indicating that the expectations for colonoscopy may be much worse than the actual experience.

In the current study, no statistically significant possible sources of heterogeneity were highlighted regarding patients’ preference. The age of the patients in both CCE and COC groups was the variable with the lowest *p*-value. As speculation, a slight tilt in preference towards COC in older patients may be explained by the higher pre-test probability of colorectal pathology, eventually leading to therapeutic COC after CCE anyway. Taking into consideration possible publication bias, the funnel plot for COC preference seems a bit skewed to the left (although mostly clustered with few outliers), whereas for CCE it is more homogenous.

Taking into account these data, when coming to tolerability parameters, surprisingly, the tolerability was consistently reported higher for CCE. It is unclear why this does not translate to higher patient preference for the modality. One explanation may be that the patient-reported advantages for COC outweigh the drawbacks and these are therefore accepted. Moreover, patients with known disease who are familiar with COC, may be more accepting of the procedure and any level of relevant discomfort, or since the tolerability is very high for both procedures, the relative higher tolerance for CCE has no actual behavioral relevance. Moreover, if the tolerance is very high for both, the risk of two examinations following a positive CCE could be considered too much of a risk.

Both examinations also require two stints of BP as the CCE report is not available immediately after excretion, enabling COC under same preparation. As BP is a documented barrier to COC and the increased cleansing regimen for CCE is a reason for preferring COC [22,32], the risk of double BP may tilt the preference towards COC, thereby playing an important role in the expression of PRO. As patient-reported procedural AE were very limited, they probably did not affect preference to a high degree, although the type of severe AE reported from COC (perforation) compared to CCE (capsule retention) is probably more likely to cause long-term effects. However, to the contrary, perforation is more common in therapeutic colonoscopies, and CCE is not suited for individuals in need of therapeutic COC [33,34].

This study comes with some limitations. The presence of small study effects for pooled preference proportion for COC (as backed up by the Egger’s tests) introduces the risk of publication bias. The I^2^ statistics indicated heterogeneous subsets of patients, and different collection methods were used in the assessments of PRO (oral interview in person, by phone or questionnaire), although no statistically significant sources were identified. Finally, for some of the included studies, only a subset of patients could be included in the meta-analysis in this review as only those subsets underwent both investigations. This introduces a risk of overrepresentation of patients with positive CCE as those excluded in the present study were discharged after negative CCE. It is expected that COC is preferred in patients with pathology as they would prefer going straight to COC since it is not possible to perform a biopsy or polypectomy with CCE.

## 5. Conclusions

The preference proportions for CCE and COC were not statistically different in this meta-analysis, with an estimated 52% preferring CCE and 45% preferring COC. Procedural AE are rare in both CCE and COC. The tolerability for CCE is generally reported higher or equal to that of COC. As a reminder for future studies, it should be emphasized to include both patients with and without positive CCE/COC to be able to estimate preference proportions for the general population.

## Figures and Tables

**Table 1:** Study characteristics.

Author—Year	Country	Study Type	PR CCE Preference	PR COC Preference	PR AE	PR Tolerability
Adrian-De-Ganzo 2015 [18]	Spain	RCT	Yes	Yes	Yes	No
Alvarez-Urturi 2017 [19]	Spain	OBS	Yes	Yes	Yes	Yes
Brechmann 2016 [20]	Germany	OBS	Yes	Yes	Yes	No
Bruining 2020 [21]	USA, Austria, Israel	OBS	Yes	Yes	No	No
Cash 2020 [22]	USA	RCT	Yes	Yes	Yes	No
D’Haens 2015 [23]	Netherlands, Belgium	OBS	Yes	No	Yes	Yes
Eliakim 2010 [24]	Israel	OBS	No	No	Yes	No
Fiorillo 2020 [25]	France	OBS	No	No	Yes	Yes
Groth 2012 [26]	Germany	OBS	Yes	Yes	Yes	No
Meister 2013 [27]	Germany	OBS	No	No	Yes	Yes
Pilz 2010 [28]	Switzerland	OBS	Yes	Yes	Yes	No
Shi 2017 [29]	Hong Kong	OBS	Yes	No	Yes	Yes
Thygesen 2019 [11]	Denmark	OBS	No	No	Yes	Yes
Voska 2019 [30]	Czech Republic	OBS	Yes	Yes	Yes	No

Abbreviations: AE, adverse events; CCE, colon capsule endoscopy; COC, conventional optical colonoscopy; OBS, observational; PR, patient-reported; RCT, randomized controlled trial.

**Table 2:** Univariate meta-regressions.

**Colon Capsule Endoscopy**				
Variable	Coefficient	Lower CI 95%	Upper CI 95%	*p*-value
Year published (continuous)	−0.003	−0.081	0.074	0.936
Year published (categorical, before vs. after 2016)	−0.058	−0.517	0.401	0.804
Sample size (continuous)	−0.002	−0.004	0.001	0.319
Study type (RCT vs. OBS)	0.116	−0.433	0.665	0.679
Centers (multi vs. single)	−0.078	−0.554	0.397	0.746
Modality of data collection (oral vs. questionnaire)	−0.209	−0.660	0.241	0.362
Age mean (continuous)	−0.023	−0.046	<0.001	0.054
Male percentage (continuous)	−0.013	−0.042	0.015	0.366
Completion rate (continuous)	−0.003	−0.024	0.018	0.772
Completion rate (categorical)	−0.018	−0.481	0.444	0.938
Capsule type (CCE2 vs. CCE1)	0.190	−0.363	0.742	0.501
Capsule type (PillCam Crohn^®^ vs. CCE1)	0.142	−0.709	0.994	0.743
Patient group (symptomatic vs. screening)	−0.045	−0.856	0.766	0.913
Patient group (chronic illness vs. screening)	0.301	−0.163	0.764	0.204
Evaluation of study (STROBE/Cochrane)	0.040	−0.520	0.601	0.887
**Colonoscopy**				
Variable	Coefficient	Lower CI 95%	Upper CI 95%	*p*-value
Year published (continuous)	0.010	−0.063	0.083	0.797
Year published (categorical, before vs. after 2016)	0.063	−0.487	0.612	0.823
Sample size (continuous)	0.002	−0.002	0.005	0.370
Study type (RCT vs. OBS)	−0.345	−0.879	0.189	0.206
Centers (multi vs. single)	−0.017	−0.609	0.575	0.955
Modality of data collection (oral vs. questionnaire)	−0.024	−0.613	0.565	0.937
Age mean (continuous)	0.022	−0.009	0.053	0.155
Male percentage (continuous)	0.004	−0.024	0.032	0.772
Completion rate (continuous)	0.057	−0.035	0.150	0.223
Completion rate (categorical)	0.006	−0.632	0.644	0.985
Patient group (symptomatic vs. screening)	−0.267	−1.103	0.568	0.531
Patient group (chronic illness vs. screening)	−0.345	−1.033	0.343	0.326
Evaluation of studies (STROBE/Cochrane)	0.036	−0.533	0.606	0.900

Abbreviations: CCE, colon capsule endoscopy; CI, confidence interval; OBS, observational; RCT, randomized controlled trial.

## Data Availability

All data in this study were obtained from already published material in scientific journals, referenced in the paper, and can be obtained by any individual with access to these.

## Appendix A

### Appendix A.1. PICO

Question: Are patient-reported outcomes for colon capsule endoscopy (CCE) superior to colonoscopy?

P: Patient subjected to either colonoscopy or/and CCE

I: Colon capsule endoscopy

C: Colonoscopy

O: All patient-reported outcomes e.g., pain, satisfaction, tolerability and days off from work.

**Table A1:** Pico scheme search terms.

Investigation	Comparator	Outcome
Capsule camera* Wireless camera* Wireless camera endoscop* WCE CCE Colon capsule endoscop* PillCam* Pill Cam* Camera pill* Capsule endoscop* Endoscop*, Capsule Wireless Capsule Endoscop* Capsule Endoscop*, Wireless Endoscop*, Wireless Capsule Video Capsule Endoscop* Capsule Endoscop*, Video Capsule Endoscopy, Video Endoscop*, Video Capsule Pan-enteric capsule endoscop*	Colonoscopy(MESH) Colonoscop* Colonoscopic Surgical Procedure* Procedure*, Colonoscopic Surgical Surgical Procedure*, Colonoscopic Surger*, Colonoscopic Surgical Procedure*, Colonoscopic Colonoscopic Surger* Colonoscopes(MESH) Colonoscope*	Patient-related outcome Patient acceptance of health care(MESH) Health Care Utilization Utilization, Health Care Patient Acceptance of Healthcare Healthcare Patient Acceptance* Nonacceptor* of Health Care Care Nonacceptor*, Health Health Care Nonacceptor* Health Care Seeking Behavior Acceptor* of Health Care Care Acceptor*, Health Health Care Acceptor* Health Care Acceptabili* Acceptability of Healthcare Healthcare Acceptabilit* Patient satisfaction(MESH) Patient preference(MESH) Patient Preference*. patient Patient comfort(MESH) Comfort, patient Comfort care Care, comfort Patient Confidence Tolerance Compliance Preference Patient-reported treatment outcome Patient-reported patientreported Self-reported Selfreported Interview* Questionnaire* Adverse event* Minor complication* Major complication* Complication* Adverse effect* Adverse reaction* PROM*

* represents any group of characters including no character.

### Appendix A.2. Search Strings

PubMed/WES:

((Capsule camera* OR Wireless camera* OR Wireless camera endoscop* OR WCE OR CCE OR Colon capsule endoscop* OR PillCam* OR Pill Cam* OR Camera pill* OR Capsule endoscop* OR Endoscop*, Capsule OR Wireless Capsule Endoscop* OR Capsule Endoscop*, Wireless OR Endoscop*, Wireless Capsule OR Video Capsule Endoscop* OR Capsule Endoscop*, Video OR Capsule Endoscopy, Video OR Endoscop*, Video Capsule OR Pan-enteric capsule endoscop*) AND (Colonoscopy OR Colonoscop* OR Colonoscopic Surgical Procedure* OR Procedure*, Colonoscopic Surgical OR Surgical Procedure*, Colonoscopic OR Surger*, Colonoscopic OR Surgical Procedure*, Colonoscopic OR Colonoscopic Surger* OR Colonoscopes OR Colonoscope*) AND (Patient-related outcome OR Patient acceptance of health care OR Health Care Utilization OR Utilization, Health Care OR Patient Acceptance of Healthcare OR Healthcare Patient Acceptance* OR Nonacceptor* of Health Care OR Care Nonacceptor*, Health OR Health Care Nonacceptor* OR Health Care Seeking Behavior OR Acceptor* of Health Care OR Care Acceptor*, Health OR Health Care Acceptor* OR Health Care Acceptabili* OR Acceptability of Healthcare OR Healthcare Acceptabilit* OR Patient satisfaction OR Patient preference OR Patient Preference* OR Patient comfort OR Comfort, patient OR Comfort care OR Care, comfort OR Patient Confidence OR Tolerance OR Compliance OR Preference OR Patient-reported treatment outcome OR Patient-reported OR patientreported OR Self-reported OR Selfreported OR Interview* OR Questionnaire* OR Adverse event* OR Minor complication* OR Major complication* OR Complication* OR Adverse effect* OR Adverse reaction* OR PROM*))

Embase:

((“Capsule camera*” or “Wireless camera*” or “Wireless camera endoscop*” or “WCE” or “CCE” or “Colon capsule endoscop*” or “PillCam*” or “Pill Cam*” or “Camera pill*” or “Capsule endoscopy/” or “Capsule endoscope/” or “Capsule endoscop*” or “Endoscop*, Capsule” or “Wireless Capsule Endoscop*” or “Capsule Endoscop*, Wireless” or “Endoscop*, Wireless Capsule” or “Video Capsule Endoscop*” or “Capsule Endoscop*, Video” or “Capsule Endoscopy, Video” or “Endoscop*, Video Capsule” or “Pan-enteric capsule endoscop*”) and (“Colonoscop*” or “Colonoscopic Surgical Procedure*” or “Procedure*, Colonoscopic Surgical” or “Surgical Procedure*, Colonoscopic” or “Surger*, Colonoscopic” or “Surgical Procedure*, Colonoscopic” or “Colonoscopic Surger*” or “Colonoscope*” or “Colonoscope/” or “Colonoscopy/”) and (“Patient-related outcome” or “Health Care Utilization” or “Utilization, Health Care” or “Patient Acceptance of Healthcare” or “Healthcare Patient Acceptance*” or “Nonacceptor* of Health Care” or “Care Nonacceptor*, Health” or “Health Care Nonacceptor*” or “Health Care Seeking Behavior” or “Acceptor* of Health Care” or “Care Acceptor*, Health” or “Health Care Acceptor*” or “Health Care Acceptabili*” or “Acceptability of Healthcare” or “Healthcare Acceptabilit*” or “Patient Preference*. patient” or “Comfort, patient” or “Comfort care” or “Care, comfort” or “Patient Confidence” or “Tolerance” or “Compliance” or “Preference” or “Patient-reported treatment outcome/ “ or “Patient-reported Patientreported outcome/” or “Self-reported” or “Selfreported” or “Interview/” or “Questionnaire/” or “Adverse event/” or “Complication/” or “Adverse effect*” or “Adverse reaction*” or “PROM*” or “Patient-reported outcome/” or “Patient attitude/” or “Health Care Utilization/” or “Patient assessment/” or “Patient satisfaction/” or “Patient preference/” or “Patient comfort/”))

## Appendix B

**Table A2:** Study evaluation of transparency and completeness.

Author, Year	Checklist	Evaluation
Alvarez-Urturi, 2017	STROBE	Medium
Brechmann, 2016	STROBE	Medium
Bruining, 2020	STROBE	High
D’Haens, 2015	STROBE	High
Eliakim, 2010	STROBE	High
Fiorillo, 2020	STROBE	High
Groth, 2012	STROBE	Medium
Meister, 2013	STROBE	Medium
Pilz, 2010	STROBE	High
Shi, 2017	STROBE	High
Thygesen, 2019	STROBE	High
Voska, 2019	STROBE	High
Adrian-De-Ganzo, 2015	RoB	High (low risk of bias)
Cash, 2020	RoB	High (low risk of bias)
